# Amelioration of Atopic Dermatitis by Frankincense Oil Extract Is Associated with TRPV3 Regulation and Cutaneous Inflammation Suppression

**DOI:** 10.3390/ijms27157061

**Published:** 2026-08-06

**Authors:** Gang-Ning Tan, Wen-Sheng Zhang, Yu-Sang Li, He-Bin Tang

**Affiliations:** Laboratory of Hepatopharmacology and Ethnopharmacology, School of Pharmaceutical Sciences, South-Central Minzu University, No. 182, Minyuan Road, Wuhan 430074, China; 2022110485@mail.scuec.edu.cn (G.-N.T.); 2023110449@mail.scuec.edu.cn (W.-S.Z.)

**Keywords:** frankincense, atopic dermatitis, TRPV3, inflammatory microenvironment

## Abstract

In traditional Chinese medicine, frankincense is widely recognized for its anti-inflammatory and immunomodulatory capacities and has been conventionally applied to manage multiple chronic inflammatory skin disorders. Nevertheless, its therapeutic potency and molecular mechanisms against atopic dermatitis (AD) remain poorly clarified. This study aimed to explore the protective efficacy of frankincense oil extract (FOE) and its active constituents in AD mouse models, thereby clarifying underlying regulatory mechanisms. Two classic AD models induced by 2,4-dinitrochlorobenzene (DNCB) and carvacrol were established to evaluate the therapeutic performance of FOE and its bioactive components. Hematoxylin–eosin and toluidine blue staining were applied to characterize lesional histopathological alterations, immunohistochemistry was used to detect expression profiles of TRPV3, β-catenin, and COX-2 in skin lesions, and calcium fluorescence imaging was applied to monitor TRPV3-mediated intracellular calcium dynamics. The results showed that FOE markedly alleviated typical AD-like manifestations, reducing inflammatory injury, ear edema and splenomegaly in DNCB- and carvacrol-challenged mice. Histological evaluation confirmed that FOE improved pathological lesions, mitigated epidermal hyperplasia, and reduced mast cell infiltration. Meanwhile, FOE remodeled the abnormal expression patterns of TRPV3, β-catenin, and COX-2 triggered by AD stimulation. Additionally, FOE effectively regulated carvacrol-evoked calcium influx mediated by TRPV3 activation. Collectively, FOE and its active components exert anti-inflammatory effects, restraining epidermal over-proliferation and ameliorating epidermal structural disorders, thereby supporting the restoration of epidermal tissue morphology. Such benefits are attributed to modulating TRPV3 activity and remodeling the cutaneous inflammatory microenvironment. This work highlights FOE as a promising novel candidate for atopic dermatitis intervention.

## 1. Introduction

Atopic dermatitis (AD), also termed atopic eczema, is a common chronic inflammatory skin disorder characterized by xerosis, eczematous lesions, aberrant immune response, and compromised skin barrier function [1,2]. Globally, AD affects approximately 15–25% of children and 3–7% of adults [3,4], representing a growing public health concern. The complex pathogenesis of AD involves multiple regulatory factors and signaling cascades. Accumulating evidence indicates that T cell-mediated immune dysfunction, skin barrier impairment [5], inflammatory mediators such as histamine and cyclooxygenase-2 (COX-2), aberrant Wnt/β-catenin signaling [6], and excessive activation of transient receptor potential vanilloid 3 (TRPV3) [7] are closely implicated in AD progression. As a natural skin sensitizer and specific TRPV3 agonist, topical exposure to carvacrol triggers typical AD-like manifestations, including pruritus, epidermal hyperplasia, and cutaneous inflammation [8,9].

Current clinical interventions for AD mainly include topical corticosteroids, calcineurin inhibitors, antihistamines, and biological agents [10], which primarily target immune disturbance and inflammatory response. However, limited therapeutic efficacy, long-term adverse reactions, and progressive drug resistance restrict their long-term application [11], highlighting an urgent demand for safer alternative strategies. As recorded in the *Chinese Pharmacopoeia*, frankincense exerts multiple pharmacological properties, including detumescence, pain relief, blood circulation promotion [12], and tissue repair, and has been conventionally adopted for various chronic inflammatory skin disorders [13].

Our previous studies have demonstrated that frankincense oil extract (FOE) and its major active constituents (1-octanol, α-pinene, and linalool) exert prominent analgesic and anti-inflammatory effects [14]. Moreover, FOE alleviates cutaneous inflammation and facilitates wound healing by restoring the aberrant expression of β-catenin and COX-2 [15,16]. Building upon these preliminary findings, we hypothesized that FOE may ameliorate AD-associated lesions and symptoms through similar regulatory patterns. Nevertheless, the therapeutic potential and detailed molecular mechanisms of FOE against AD remain poorly defined in modern pharmacological research. The present study was designed to explore the therapeutic effects of topically applied FOE in DNCB- and carvacrol-induced AD mouse models, with the aim of further elucidating the underlying regulatory mechanisms for FOE in AD intervention.

## 2. Results

### 2.1. Commonly Used Clinical Drugs and Related Inhibitors Alleviated DNCB-Induced AD-like Skin Inflammation

**Figure 1:**
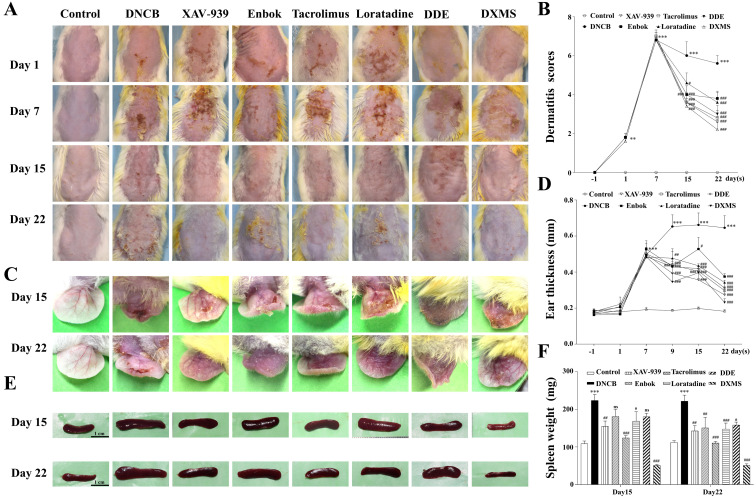
The effects of clinically used drugs and related inhibitor treatment on DNCB-induced AD mice. (**A**) Dermatitis characteristics of the back skin at different time points in each group. (**B**) Dynamic dermatitis severity score. (**C**) Representative photographs of ear swelling. (**D**) Ear thickness. (**E**) Representative photographs of mouse spleens. (**F**) Spleen weight. Data were expressed as mean ± SEM and analyzed by two-way ANOVA, n = 10. Scale bar, 1 cm. DXMS: Dexamethasone; DDE: diclofenac diethylamine emulgel. ** *p* < 0.01, *** *p* < 0.001 compared with the control group; # *p* < 0.05, ## *p* < 0.01, ### *p* < 0.001 compared with the model group; ns, not significant.

We selected commonly used clinical anti-atopic dermatitis (AD) agents, including dexamethasone, tacrolimus, loratadine, and enbok, together with the β-catenin inhibitor XAV-939 and COX inhibitor DDE. As shown in Figure 1, repetitive topical DNCB stimulation induced severe AD-like lesions, characterized by erythema, dryness, scaling, swelling, and ulceration, with the most serious symptoms peaking on day 7. Compared with the DNCB group, each treatment group displayed distinct amelioration of skin lesions and a marked decline in dermatitis scores. Dexamethasone, tacrolimus, and XAV-939 yielded the most pronounced therapeutic benefits. Moreover, DNCB markedly increased ear thickness and spleen weight, while all interventions alleviated these abnormalities to different degrees. Long-term dexamethasone treatment caused obvious splenic atrophy, with splenic parameters even lower than blank control levels. Collectively, these data indicate that the Wnt/β-catenin cascade, alongside COX-2, IL-8, and histamine-mediated inflammation, participates in AD pathogenesis. Targeting these pathways can effectively relieve AD manifestations, whereas suboptimal efficacy and prominent adverse effects restrict the long-term application of clinical drugs. These observations lay a solid foundation for the subsequent exploration of natural candidate therapeutic agents for AD.

### 2.2. FOE and Its Active Ingredients Alleviated DNCB-Induced AD-like Skin Inflammation

**Figure 2:**
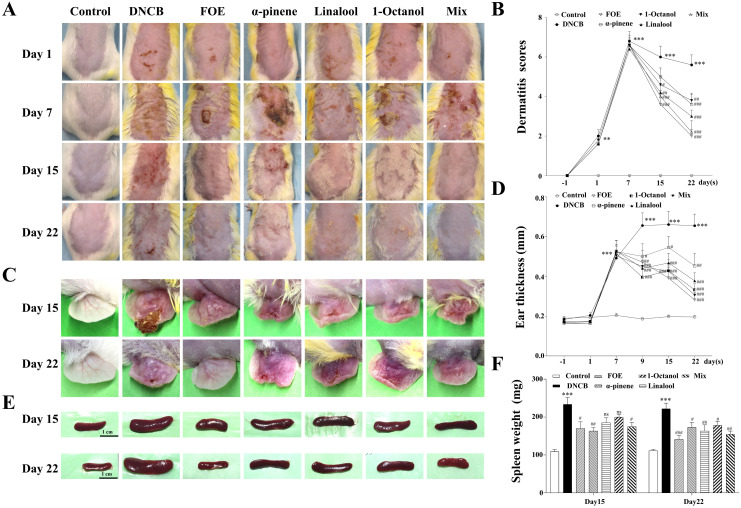
The effects of FOE treatment on DNCB-induced AD mice. (**A**) Dermatitis characteristics of the back skin at different time points in each group. (**B**) Dynamic changes in dermatitis severity scores. (**C**) Representative photographs of ear swelling. (**D**) Ear thickness. (**E**) Representative photographs of mouse spleens. (**F**) Spleen weight. Data were expressed as mean ± SEM and analyzed by two-way ANOVA, n = 10. Scale bar, 1 cm. Mix: mixture (linalool + α-pinene + 1-octanol). ** *p* < 0.01, *** *p* < 0.001 compared with the control group; # *p* < 0.05, ## *p* < 0.01, ### *p* < 0.001 compared with the model group; ns, not significant.

To assess the anti-AD effects of FOE against DNCB-triggered skin damage, multiple phenotypic indicators were evaluated. As shown in Figure 2, obvious AD-like manifestations, including xerosis, erythema, hemorrhage, edema, and ulceration, emerged in all groups except the control on day 7 after continuous DNCB stimulation. The DNCB model group exhibited severe erythema, vesiculation, crusting, and edema on day 15, with extensive inflammatory lesions remaining persistent on day 22 without spontaneous remission. Compared with the DNCB group, topical FOE markedly ameliorated skin erythema, edema, crusting and vesiculation, accompanied by reduced dermatitis scores (day 15: 40 ± 8.5%, *p* < 0.001; day 22: 61.5 ± 6.1%, *p* < 0.001), ear thickness (day 15: 41.5 ± 9.3%, *p* < 0.001; day 22: 56.3 ± 3.7%, *p* < 0.001) and spleen weight (day 15: 26 ± 7.6%, *p* < 0.05; day 22: 36.9 ± 4.4%, *p* < 0.001). Similarly, α-pinene, linalool, 1-octanol, and their mixture notably relieved AD-like lesions and reduced dermatitis scores, ear swelling, and splenic enlargement. Collectively, FOE and its active constituents can effectively alleviate DNCB-induced atopic dermatitis-like inflammation in mice.

### 2.3. Histopathological Improvements of AD Skin Lesions by FOE, Its Active Components, Clinical Agents, and Related Inhibitors

To further explore the protective efficacy of FOE on AD progression, histological alterations were assessed via HE and toluidine blue staining at the mid (day 15) and late (day 22) repair stages (Supplementary Figures S1 and S2). Compared with the control group, DNCB-challenged mice exhibited obvious epidermal thickening, corneal hyperkeratosis, and extensive dermal infiltration of eosinophils and mast cells on day 15. Such pathological abnormalities were further exacerbated in the DNCB group on day 22. Relative to the model group, topical FOE intervention greatly mitigated AD-associated histopathological damage, accompanied by reduced pathological scores (day 15: 59 ± 4.8%, *p* < 0.001; day 22: 73.2 ± 4.6%, *p* < 0.001), attenuated epidermis (day 15: 60 ± 3.2%, *p* < 0.001; day 22: 69.9 ± 3%, *p* < 0.001), and diminished mast cell infiltration (day 15: 45.4 ± 3.7%, *p* < 0.001; day 22: 52.7 ± 3.7%, *p* < 0.001). Similarly, α-pinene, linalool, 1-octanol, and their mixture also markedly alleviated cutaneous pathological injuries.

We further evaluated the histological benefits of conventional clinical drugs and targeted inhibitors. XAV-939, DDE, dexamethasone, tacrolimus, loratadine, and enbok all effectively relieved AD histopathological lesions, among which dexamethasone exerted the strongest therapeutic potency. Notably, long-term dexamethasone exposure induced obvious skin atrophy and epidermal thinning.

### 2.4. FOE, Its Active Components, Clinical Agents, and Related Inhibitors Modulate Aberrant β-Catenin Expression in AD Lesions

**Figure 3:**
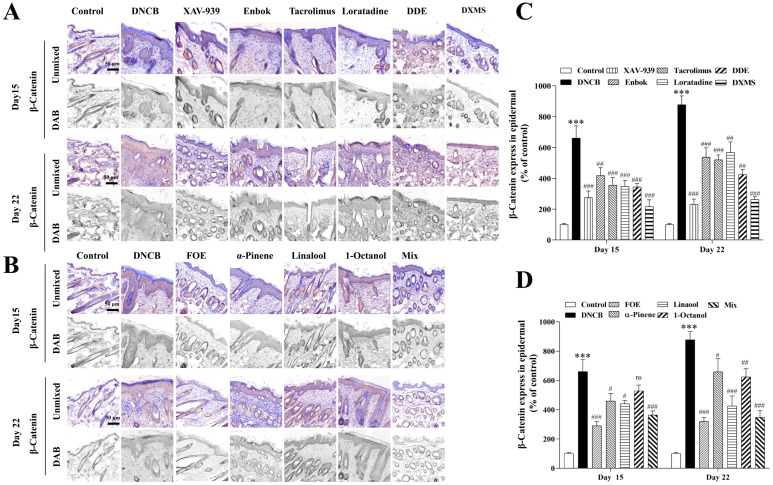
Immunohistochemical staining of β-catenin in mouse skin tissues at different time points and their quantitative analysis. Representative images of immunohistochemical staining for β-catenin in the skin of AD mice in the clinically used drugs and related inhibitor groups (**A**) and the FOE and its active constituent groups (**B**). Quantitative multispectral image of the β-catenin expression of clinically used drugs and related inhibiting groups (**C**) and in the FOE and its active ingredient groups (**D**). Data were expressed as mean ± SEM and analyzed by two-way ANOVA, n = 10. Scale bar, 50 µm. *** *p* < 0.001 compared with the control group; # *p* < 0.05, ## *p* < 0.01, ### *p* < 0.001 compared with the model group; ns, not significant.

β-Catenin serves as a core signaling molecule regulating keratinocyte proliferation and cutaneous inflammation [17]. Excessive β-catenin accumulation promotes epidermal hyperplasia and thus facilitates AD progression [18]. Immunohistochemical staining results are shown in Figure 3A. Relative to the control group (day 15: 100 ± 8%; day 22: 100 ± 7%), DNCB stimulation markedly elevated epidermal β-catenin abundance (day 15: 660 ± 81% of control, *p* < 0.001; day 22: 877 ± 57% of control, *p* < 0.001). As a specific β-catenin inhibitor, XAV-939 reversed such abnormal upregulation (day 15: 197 ± 33% of control, *p* < 0.001; day 22: 232 ± 33% of control, *p* < 0.001). These data confirm the crucial involvement of Wnt/β-catenin signaling in AD pathogenesis and that targeting its abnormal accumulation ameliorates AD-like lesions. Similarly, DDE, dexamethasone, tacrolimus, loratadine, and enbok all relieved aberrant β-catenin elevation to different extents.

To further clarify the protective mechanism of FOE, β-catenin expression in skin tissues was detected (Figure 3B). FOE intervention effectively suppressed abnormal β-catenin accumulation (day 15: 290 ± 31% of control, *p* < 0.001; day 22: 320 ± 27% of control, *p* < 0.001), compared with the DNCB model (day 15: 656 ± 78%; day 22: 867 ± 55%). In line with this, linalool, α-pinene, 1-octanol, and their combination also alleviated abnormal β-catenin expression.

Collectively, FOE and its bioactive constituents effectively rectified dysregulated β-catenin profiles in AD skin tissues.

### 2.5. FOE, Its Active Components, Clinical Agents, and Inhibitors Regulate Aberrant COX-2 Expression in AD Skin

**Figure 4:**
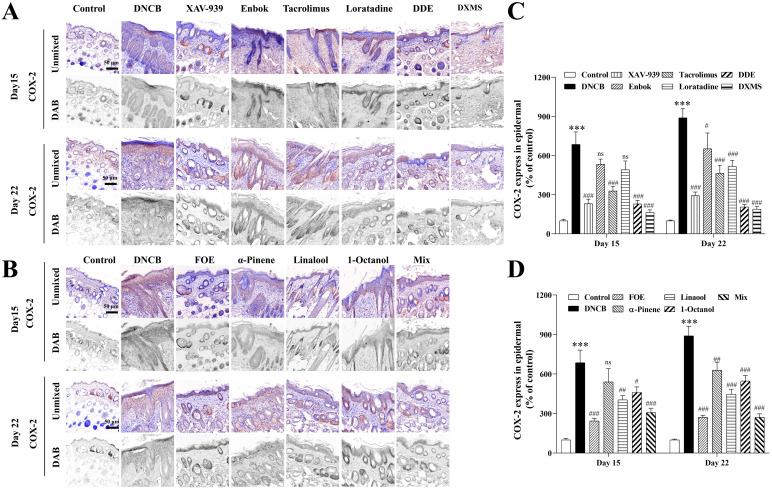
Immunohistochemical staining of COX-2 in mouse skin tissues at different time points and their quantitative analysis. Representative images of immunohistochemical staining for COX-2 in the skin of AD mice in the clinically used drugs and related inhibitor groups (**A**) and the FOE and its active constituent groups (**B**). Quantitative multispectral image of the COX-2 expression of clinically used drugs and related inhibiting groups (**C**) and in the FOE and its active ingredient groups (**D**). Data were expressed as mean ± SEM and analyzed by two-way ANOVA, n = 10. Scale bar, 50 µm. *** *p* < 0.001 compared with the control group; # *p* < 0.05, ## *p* < 0.01, ### *p* < 0.001 compared with the model group; ns, not significant.

COX-2 is a pivotal inflammation-related enzyme secreted by epithelial cells and functions as a core driver of cutaneous inflammatory responses in AD [19]. Immunohistochemical staining results are shown in Figure 4A. Relative to the control group (day 15: 100 ± 11%; day 22: 100 ± 7%), DNCB challenge markedly increased COX-2 expression in epidermal keratinocytes (day 15: 678 ± 91% of control, *p* < 0.001; day 22: 871 ± 79% of control, *p* < 0.001). As a selective COX inhibitor, DDE greatly reversed such abnormal COX-2 elevation (day 15: 189 ± 10% of control, *p* < 0.001; day 22: 166 ± 14% of control, *p* < 0.001). These results confirm that aberrant COX-2 activation contributes to AD pathogenesis and that restoring its dysregulated expression serves as a critical strategy for AD alleviation. Similarly, XAV-939, dexamethasone, tacrolimus, loratadine, and enbok all alleviated excessive COX-2 accumulation to varying degrees.

COX-2 expression in FOE-intervened tissues was further detected (Figure 4B). Compared with the DNCB group (day 15: 686 ± 95% of control; day 22: 899 ± 73% of control), FOE treatment prominently reduced abnormal COX-2 abundance in keratinocytes (day 15: 243 ± 21% of control, *p* < 0.001; day 22: 272 ± 15% of control, *p* < 0.001). In parallel, linalool, α-pinene, 1-octanol, and their mixture exerted consistent regulatory effects on rectifying dysregulated COX-2 profiles.

Together, FOE and its bioactive constituents improve cutaneous inflammation and excessive epidermal proliferation via rectifying the abnormal expression of β-catenin and COX-2 in AD lesions.

### 2.6. FOE and Its Active Components Modulate Aberrant TRPV3 Expression in AD Skin

TRPV3 is abundantly expressed in the epidermis and keratinocytes of AD lesional skin [7]. Excessive TRPV3 activation induces skin inflammation, pruritus [20], keratinocyte hyperproliferation [21], and skin barrier disruption [22], making TRPV3 a potential therapeutic target for AD intervention [23].

**Figure 5:**
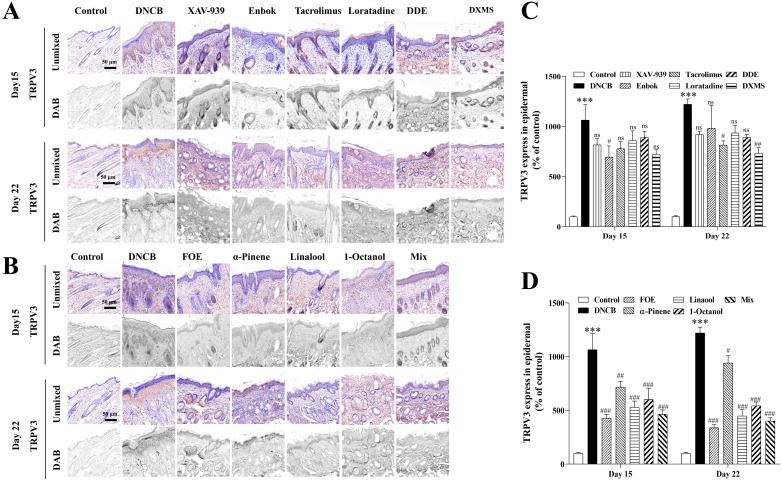
Immunohistochemical staining of TRPV3 in mouse skin tissues at different time points and their quantitative analysis. Representative images of immunohistochemical staining for TRPV3 in the skin of AD mice in the clinically used drugs and related inhibiting groups (**A**) and the FOE and its active constituent groups (**B**). Quantitative multispectral image of the TRPV3 expression of clinically used drugs and related inhibitor groups (**C**) and in the FOE and its active ingredient groups (**D**). Data were expressed as mean ± SEM and analyzed by two-way ANOVA, n = 10. Scale bar, 50 µm. *** *p* < 0.001 compared with the control group; # *p* < 0.05, ## *p* < 0.01, ### *p* < 0.001 compared with the model group; ns, not significant.

Immunohistochemical staining results are illustrated in Figure 5A. Relative to the control group (day 15: 100 ± 7%; day 22: 100 ± 10%), DNCB stimulation strongly increased epidermal TRPV3 abundance (day 15: 1065 ± 154% of control, *p* < 0.001; day 22: 1221 ± 54% of control, *p* < 0.001). Notably, conventional clinical drugs, as well as β-catenin and COX pathway inhibitors, exerted no obvious regulatory effect on dysregulated TRPV3 expression in AD mice.

To further clarify whether FOE and its constituents ameliorate AD lesions by targeting TRPV3 signaling, immunohistochemical detection was conducted. As shown in Figure 5B, TRPV3 levels in the FOE-treated group were markedly restored (day 15: 490 ± 38% of control, *p* < 0.001; day 22: 378 ± 28% of control, *p* < 0.001) compared with the DNCB model (day 15: 1007 ± 121% of control; day 22: 1171 ± 44% of control). Similarly, α-pinene, linalool, 1-octanol, and their mixture alleviated TRPV3 dysregulation in keratinocytes. These findings suggest that FOE and its bioactive constituents improve AD lesions through rectifying abnormal TRPV3 expression profiles.

### 2.7. The TRPV3 Inhibitor Dyclonine Ameliorates Carvacrol-Induced Dermatitis

**Figure 6:**
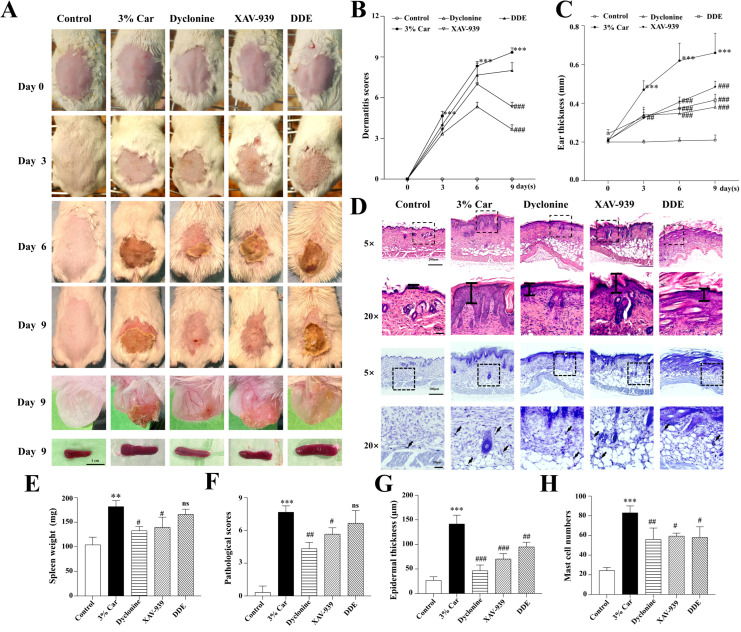
The effects of TRPV3, β-catenin and COX inhibitors treatment on carvacrol-induced AD mice. (**A**) Representative photographs of back skin clinical features, ear swelling, and spleen at various time points in each group. (**B**) Dynamic changes in dermatitis severity scores. (**C**) Ear thickness. (**D**) Representative images of HE staining and toluidine blue staining. (**E**) Spleen weight. (**F**) Pathological scores. (**G**) Epidermal thickness. (**H**) Mast cell numbers. Black line segment indicates the epidermal thickness of dorsal skin; mast cells are highlighted by black arrows. Data were expressed as mean ± SEM and analyzed by One-way or two-way ANOVA, n = 3. Scale bars: 1 cm, 200 μm, 50 μm. ** *p* < 0.01, *** *p* < 0.001 compared with the control group; # *p* < 0.05, ## *p* < 0.01, ### *p* < 0.001 compared with the model group; ns, not significant.

To further verify the pathogenic contribution of excessive TRPV3 activation, a mouse AD model was established by daily topical application of 3% carvacrol for 8 consecutive days. As shown in Figure 6, repeated carvacrol exposure induced typical AD-like manifestations, including xerosis, erythema, crusting, and edema. Compared with the carvacrol model group, dyclonine intervention markedly ameliorated skin lesions, accompanied by reduced dermatitis scores (60.7 ± 3.6%, *p* < 0.001), ear thickness (by 42.4 ± 2.3%, *p* < 0.001), spleen weight (by 27 ± 2.8%, *p* < 0.05), and pathological scores (by 40 ± 6.9%, *p* < 0.05). Epidermal thickness and mast cell numbers were also reduced by 61.7 ± 4.6% (*p* < 0.001) and 32.5 ± 8.0% (*p* < 0.01), respectively. These findings confirm that sustained TRPV3 overactivation promotes AD progression, and targeted regulation of TRPV3 activity provides a viable therapeutic option for AD. In contrast, the β-catenin inhibitor XAV-939 and COX inhibitor DDE exerted limited protective effects against carvacrol-induced dermatitis, and DDE showed nearly no observable beneficial effects.

### 2.8. FOE and Its Active Components Meliorate Carvacrol-Induced Dermatitis

**Figure 7:**
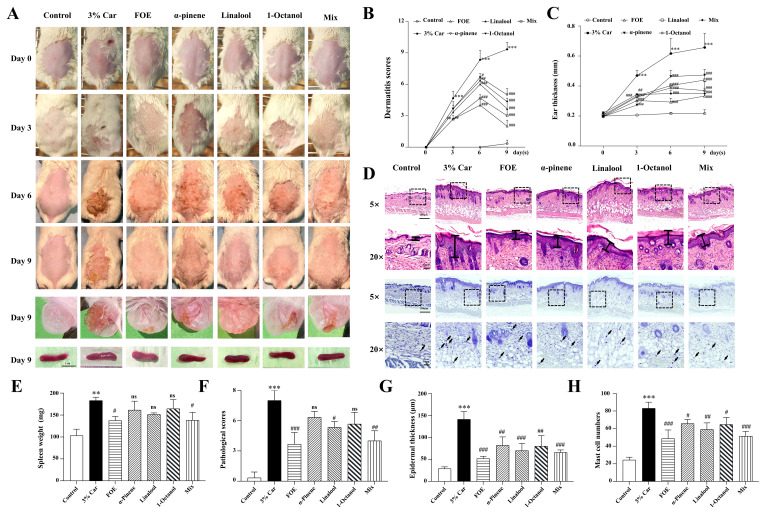
The effects of FOE treatment on carvacrol-induced AD mice. (**A**) Representative photographs of back skin clinical features, ear swelling, and spleen at various time points in each group. (**B**) Dynamic changes in dermatitis severity scores. (**C**) Ear thickness. (**D**) Representative images of HE staining and toluidine blue staining. (**E**) Spleen weight. (**F**) Pathological scores. (**G**) Epidermal thickness. (**H**) Mast cell numbers. The black line segment indicates the epidermal thickness of dorsal skin; mast cells are highlighted by black arrows. Data were expressed as mean ± SEM and analyzed by One-way or two-way ANOVA, n = 3. Scale bars: 1 cm, 200 μm, 50 μm. ** *p* < 0.01, *** *p* < 0.001 compared with the control group; # *p* < 0.05, ## *p* < 0.01, ### *p* < 0.001 compared with the model group; ns, not significant.

Given that FOE and its bioactive constituents ameliorated DNCB-induced skin damage and rectified aberrant TRPV3 expression, we further validated their therapeutic effects in a carvacrol-induced AD model. As shown in Figure 7, repeated 3% carvacrol topical stimulation induced obvious AD-like lesions in mice. Relative to the carvacrol model group, topical FOE treatment greatly alleviated skin injury, accompanied by decreased dermatitis scores (77.8 ± 6.4%, *p* < 0.001), ear thickness (47.9 ± 2.8%, *p* < 0.001), spleen weight (23.1 ± 3.1%, *p* < 0.05), and pathological scores (54.2 ± 8.3%, *p* < 0.05). Epidermal thickness and mast cell infiltration were also reduced by 64.4 ± 2.1% (*p* < 0.001) and 39.9 ± 7.0% (*p* < 0.01), respectively. Similarly, α-pinene, linalool, 1-octanol, and their mixture mitigated carvacrol-mediated cutaneous lesions to varying extents. Collectively, these data confirm that FOE and its active ingredients improve AD manifestations via regulating excessive TRPV3 activity.

### 2.9. Carvacrol Induces Aberrant Upregulation of TRPV3, β-Catenin and COX-2 in AD Skin Tissues

**Figure 8:**
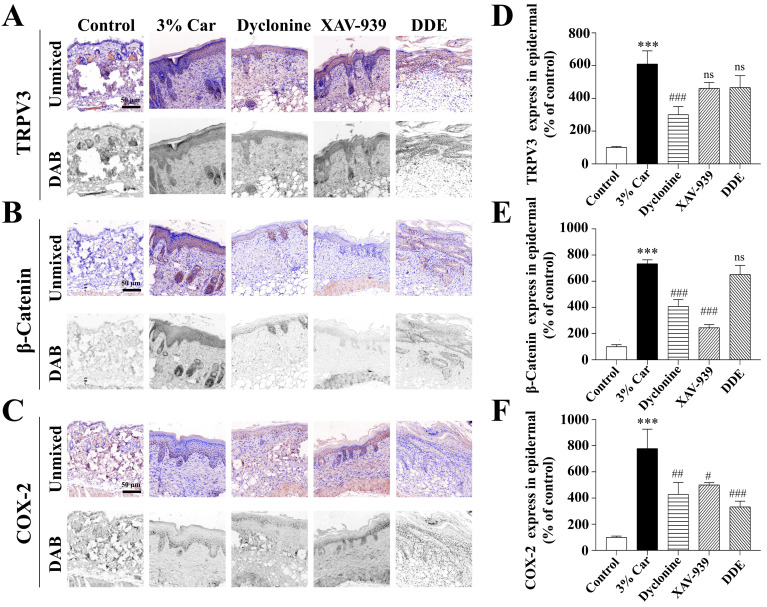
The effects of related inhibitors on TRPV3, β-catenin and COX-2 expressions in the skin tissues of AD mice. Representative images of immunohistochemical staining for TRPV3 (**A**) β-catenin (**B**) and COX-2 (**C**) in the skin of AD mice. Quantitative multispectral images of TRPV3 (**D**) β-catenin (**E**) and COX-2 (**F**) expressions. Data were expressed as mean ± SEM and analyzed by One-way ANOVA, n = 3. Scale bar, 50 µm. *** *p* < 0.001 compared with the control group; # *p* < 0.05, ## *p* < 0.01, ### *p* < 0.001 compared with the model group; ns, not significant.

To clarify the regulatory association among TRPV3, β-catenin, and COX-2, immunohistochemical analysis was performed using corresponding pathway inhibitors. As illustrated in Figure 8, carvacrol challenge markedly increased epidermal TRPV3 expression (610 ± 46% of control, *p* < 0.001) relative to the control (100 ± 4%). Dyclonine pretreatment obviously rectified such TRPV3 elevation (301 ± 28% of control, *p* < 0.001) compared with the carvacrol group. In comparison, XAV-939 and DDE exerted no obvious effects on TRPV3 abundance (462 ± 20% and 466 ± 42% of control, respectively, both *p* > 0.05).

Compared with the carvacrol group, both dyclonine and XAV-939 greatly reduced epidermal β-catenin levels (409 ± 29% and 245 ± 14% of control, *p* < 0.001). By contrast, DDE treatment failed to alter β-catenin expression (651 ± 40% of control, *p* > 0.05).

Carvacrol also induced remarkable COX-2 upregulation (779 ± 85% of control, *p* < 0.001) versus the control (100 ± 6%). Dyclonine, XAV-939 and DDE all effectively suppressed excessive COX-2 accumulation (428 ± 53%, 501 ± 10% and 315 ± 27% of control, *p* < 0.01, *p* < 0.05, *p* < 0.001, respectively).

Overall, blockade of TRPV3 signaling ameliorated the abnormal elevation of β-catenin and COX-2, while β-catenin or COX-2 inhibition failed to affect TRPV3 dysregulation. These data confirm that β-catenin and COX-2 act as downstream effectors of the TRPV3 cascade in carvacrol-induced AD lesions.

### 2.10. FOE and Its Active Components Reverse Carvacrol-Triggered Upregulation of TRPV3, β-Catenin and COX-2

**Figure 9:**
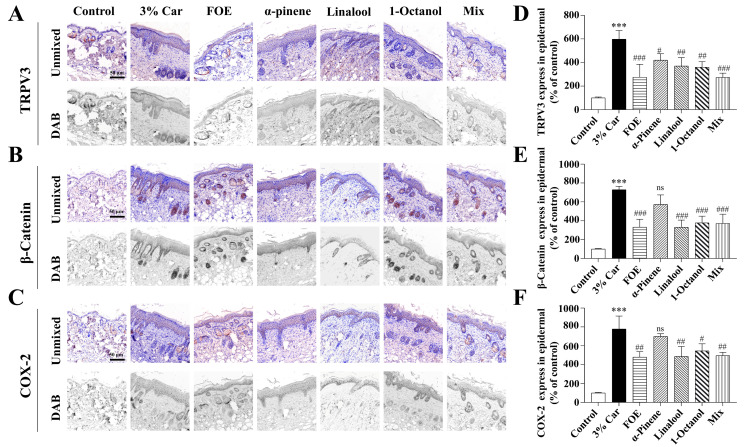
The effects of FOE on TRPV3, β-catenin and COX-2 expressions in the skin tissues of AD mice. Representative images of immunohistochemical staining for TRPV3 (**A**), β-catenin (**B**), and COX-2 (**C**) in the skin of AD mice. Quantitative multispectral images of TRPV3 (**D**), β-catenin (**E**) and COX-2 (**F**) expressions. Data were expressed as mean ± SEM and analyzed by One-way ANOVA, n = 3. Scale bar, 50 µm. *** *p* < 0.001 compared with the control group; # *p* < 0.05, ## *p* < 0.01, ### *p* < 0.001 compared with the model group; ns, not significant.

As shown in Figure 9, the expression of TRPV3, β-catenin and COX-2 was markedly reduced in the FOE group (TRPV3: 275 ± 63% of control, *p* < 0.001; β-catenin: 275 ± 63% of control, *p* < 0.001; COX-2: 480 ± 33% of control, *p* < 0.01) than in the carvacrol model group (TRPV3: 598 ± 42% of control; β-catenin: 729 ± 20% of control; COX-2: 778 ± 79% of control). Similarly, FOE-derived active constituents also downregulated the abnormal expression of these three proteins. In brief, FOE and its bioactive compounds ameliorate cutaneous inflammation and excessive epidermal proliferation through rectifying aberrant TRPV3, β-catenin and COX-2 profiles, thereby promoting the restoration of well-organized epidermal tissue architecture and alleviating AD-like lesions.

### 2.11. Effects of FOE and Its Active Components on TRPV3-Mediated Calcium Influx in Primary Keratinocytes

**Figure 10:**
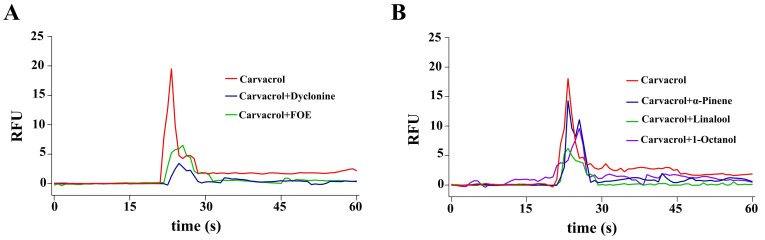
The effects of FOE and its active components on TRPV3-mediated calcium ion fluorescence intensity in primary keratinocytes. (**A**) FOE and dyclonine inhibit the TRPV3-mediated increase in intracellular calcium levels induced by 500 μM carvacrol in primary keratinocytes. (**B**) α-pinene, linalool, and 1-octanol exert similar inhibitory effects on the TRPV3-mediated intracellular calcium elevation induced by 500 μM carvacrol in primary keratinocytes.

To further clarify the regulation of TRPV3 signaling, we detected intracellular calcium dynamics in primary keratinocytes. As shown in Figure 10, 500 μM carvacrol markedly increased intracellular calcium fluorescence intensity, indicating TRPV3 activation. Both the TRPV3 inhibitor dyclonine and FOE greatly attenuated carvacrol-provoked calcium influx. The three active constituents of FOE also relieved carvacrol-induced calcium elevation to different extents. These findings confirm that FOE and its bioactive ingredients effectively modulate TRPV3 channel activity, thereby balancing TRPV3-dependent signaling in keratinocytes.

## 3. Discussion

This study evaluated the therapeutic effects and potential mechanisms of FOE and its active components against DNCB- and carvacrol-induced AD. FOE intervention markedly ameliorated typical AD-like lesions, including xerosis, erythema, edema, erosion, increased ear thickness, splenomegaly, epidermal hyperplasia, and dermal infiltration of mast cells and eosinophils. Meanwhile, FOE effectively rectified the abnormal upregulation of TRPV3, β-catenin, and COX-2 triggered by DNCB and carvacrol, suggesting great potential for the experimental treatment of AD intervention.

This work also compared the efficacy of commonly used anti-AD agents, including dexamethasone [24], tacrolimus [25], loratadine [26], and the anti-IL-8 monoclonal antibody enbok [27]. Most conventional preparations exhibit suboptimal therapeutic efficacy or obvious adverse reactions [1], which greatly limit their clinical application. Topical glucocorticoids such as dexamethasone serve as first-line therapy for moderate-to-severe atopic dermatitis, characterized by rapid onset and potent anti-inflammatory activity. In this study, dexamethasone produced the strongest therapeutic effect on DNCB-induced AD-like skin lesions, as demonstrated by phenotypic and histological results. However, dexamethasone treatment also induced evident adverse phenotypes, including epidermal thinning and splenic atrophy, as observed in Figure 1 and Supplementary Figure S1. Notably, the pronounced splenic atrophy observed by Day 22 is a typical phenotype of systemic immune suppression caused by transdermal absorption of corticosteroids. In clinical practice, prolonged or extensive application of topical glucocorticoids carries a well-recognized risk of systemic absorption, which may lead to immune organ atrophy, hypothalamic–pituitary–adrenal axis suppression and other systemic adverse events, greatly restricting long-term clinical use. Therefore, it is necessary to develop safe and efficient alternative candidates for AD intervention. Frankincense, a traditional Chinese medicine with well-documented anti-inflammatory, analgesic, and immunomodulatory properties [28], serves as a reliable natural candidate. In DNCB-induced AD models, topical administration of FOE effectively ameliorated AD-like skin lesions without triggering epidermal atrophy or splenic abnormalities, supporting its potential as a safer candidate for AD intervention.

Epidermal hyperplasia and excessive dermal infiltration of mast cells and eosinophils are core pathological hallmarks of AD [29]. FOE intervention significantly reduced epidermal thickness and attenuated the accumulation of inflammatory cells in dermal tissues. As a vital immune organ closely correlated with disease severity [30], the spleen exhibited increased weight and volume in both DNCB and carvacrol AD models, and such abnormal immune changes can be effectively reversed by FOE. These findings support that FOE exerts prominent anti-inflammatory and immunomodulatory properties in AD progression.

TRPV3 acts as a pivotal regulatory molecule in AD pathogenesis [31]. Excessive TRPV3 activation exacerbates skin inflammation, pruritus, epidermal hyperplasia, and barrier dysfunction. Mechanistically, sustained TRPV3 opening induces intracellular calcium overload, which further facilitates the release of inflammatory mediators such as TNF-α, IL-6, and IL-8, thereby amplifying cutaneous inflammatory injury and pruritus [32,33]. Accumulated evidence has demonstrated that natural TRPV3 modulators can effectively ameliorate pruritus and dermatitis [8,23].

A carvacrol-induced AD model was established based on published protocols [8], and dyclonine was used as a positive control. Dyclonine normalized TRPV3 overactivation and alleviated carvacrol-provoked skin damage, verifying that balanced TRPV3 activity is essential for AD relief. FOE exerted similar protective effects, demonstrating that the improvement of AD lesions by FOE is closely related to the regulation of overactive TRPV3 channel function.

Multiple studies have confirmed that FOE ameliorates skin inflammation via modulating the β-catenin/COX-2 signaling axis [16,34]. Inflammatory stimulation promotes nuclear translocation and accumulation of β-catenin, which further elevates COX-2 expression and PGE_2_ production [35]. This positive feedback loop continuously amplifies inflammatory responses and deteriorates chronic skin disorders. FOE blocked this pathological cycle by remodeling dysregulated β-catenin and COX-2 expression, thereby mitigating AD-related cutaneous inflammation.

Aberrant β-catenin signaling contributes to dry skin-associated chronic pruritus, and β-catenin modulation by XAV-939 alleviates epidermal hyperplasia and pruritus [36]. Increased COX-2 and PGE_2_ levels are also widely detected in clinical AD tissues and serum samples [37]. COX-2/PGE_2_ overexpression inhibits filaggrin synthesis and impairs skin barrier function, further exacerbating AD deterioration [38]. In the present study, XAV-939 exerted satisfactory anti-inflammatory effects, whereas DDE showed limited therapeutic benefits. Such discrepancies may stem from skin irritation caused by propylene glycol in DDE preparations [39]. Hence, topical formulations containing propylene glycol should be cautiously applied for AD intervention. Targeted regulation of β-catenin and COX-2 abnormalities is critical for alleviating excessive inflammation and reducing epidermal hyperproliferation. The present study did not assess the effects of FOE on disease progression after treatment cessation. Whether the remodeling of the TRPV3 cascade can induce long-term remission of AD, or whether continuous application is required to prevent relapse, will be systematically investigated in our follow-up studies. Beyond the lack of post-withdrawal long-term efficacy data, a notable limitation is that multifunctional skin barrier biomarkers were not profiled, which limited robust verification of the amelioration of disrupted epidermal morphology. To address this, we will expand the panel of detected epidermal functional indicators and perform comprehensive mechanistic analyses in future studies.

## 4. Materials and Methods

### 4.1. Materials

Frankincense (gum resin from *Boswellia carterii* Birdw., Ethiopia), identified by Prof. Xiaochuan Ye (Hubei University of Chinese Medicine, Wuhan, China), was purchased from Yinpian Factory, Guangzhou Medicine Company (Guangzhou, China). Frankincense oil extract (FOE) was analyzed using the GC-MS method, and three components were identified in FOE: 1-octanol (0.359 mg/mL), α-pinene (0.072 mg/mL), and linalool (0.0593 mg/mL). The FOE utilized in this research was sourced from previously collected samples [No. 20150313], preserved in the sampling facility of the Hepato-pharmacology and Ethnopharmacology department at the School of Pharmaceutical Sciences, South-Central Minzu University [14].

The drugs used in this study included: linalool (Aladdin; Shanghai, China); α-pinene (TCI; Shanghai, China); 2,4-dinitrochlorobenzene (Aladdin; Shanghai, China); tacrolimus ointment (Renfuchengtian Pharmaceutical Co., Ltd.; Tianmen, China); 1-octanol (Sinopharm Chemical Reagent Co., Ltd.; Shanghai, China); loratadine tablets (Haishen Tongzhou Pharmaceutical Co., Ltd.; Haikou, China); enbok (Yawei Pharmaceutical Co., Ltd., Dalian, China); diclofenac diethylamine emulgel (DDE; Novartis Pharma Co., Ltd.; Beijing, China); compound dexamethasone acetate ointment (CR Sanjiu; Beijing, China); XAV-939 (Glpbio, Montclair, CA, USA); dyclonine hydrochloride (Aladdin, Shanghai, China); and carvacrol (Sigma, Shanghai, China). The primary antibodies applied in this study included anti-β-catenin (Abcam, Shanghai, China), anti-COX-2 (Cell Signaling Technology, Danvers, MA, USA), and anti-TRPV3 (Invitrogen, Carlsbad, CA, USA). Analytical kits comprised a Hematoxylin and Eosin (HE) staining kit (Njjcbio, Nanjing, China) and a Toluidine blue staining kit (Leagene, Beijing, China). The reagents used in the cell experiments included DMEM (Servicebio, Wuhan, China), RPMI-1640 (Servicebio, Wuhan, China), Dispase II (Merck, Darmstadt, Germany), 0.25% trypsin digestion solution (Servicebio, Wuhan, China), Fluo-4 (Invitrogen, USA), and FBS (Nova Pharmaceutical Technology Co., Ltd.; Shanghai, China).

### 4.2. Animal Models

Eight-week-old male Kunming mice (25–30 g) were purchased from the Laboratory Animal Center, Wuhan, Hubei, China, and acclimated for 7 days in a controlled, pathogen-free environment, with unrestricted access to food and water. The housing conditions maintained a 12 h light–dark cycle, and the temperature was regulated between 22–25 °C.

A total of 150 male mice were randomly divided into 15 groups (n = 10). Groups 1–8 were treated with vehicle, DNCB, XAV-939 (Wnt/β-catenin inhibitor, 0.5 mg/mL, 5 mg/kg/d), DDE (COX inhibitor, 15 mL/kg), loratadine (histamine inhibitor, 0.063 g/mL, 10 mg/kg/d), tacrolimus, anti-IL-8 monoclonal antibody, and dexamethasone in sequence. Groups 9–15 were given vehicle, DNCB, FOE (150 mg/mL), α-pinene (0.072 mg/mL), linalool (0.0593 mg/mL), 1-octanol (0.0359 mg/mL), and their mixed formulation.

One day before modeling, the mice’s dorsal hair was shaved to a 3 cm × 4 cm hairless area. Except for the vehicle group, mice were sensitized with 2% DNCB solution (acetone/olive oil = 3:1, *v*/*v*) on depilated dorsal skin and the right ear on day 0 and day 3, with dosages of 120 μL and 30 μL, respectively. Repeated 0.5% DNCB stimulation was performed on days 6, 9, 12, 15, 18, and 21 [30]. The vehicle group was treated with an equal volume of solvent. All drug interventions started on day 7 and were administered once daily for 15 consecutive days. Half of the mice in each group were euthanized on days 15 and 22.

Another 36 male mice were randomized into 12 groups (n = 3). Groups 1–5 received vehicle, carvacrol, dyclonine (TRPV3 inhibitor, 0.5 mg/mL, 5 mg/kg/d), XAV-939, and DDE. Groups 6–12 were treated with vehicle, carvacrol, FOE, α-pinene, linalool, 1-octanol, and their compound mixture (same dosages as above). The carvacrol-induced AD model was established with minor modifications [23]. Carvacrol was dissolved in 50% ethanol-normal saline. Dorsal hair removal was performed using the same procedure as the DNCB model. AD-like lesions were induced by daily topical application of 3% carvacrol on the dorsal skin and ears for 8 consecutive days. All drugs were administered 30 min before carvacrol treatment per day, and all mice were euthanized on day 9.

### 4.3. Assessment of Dermatitis Score, Ear Swelling, and Spleen Weight

Digital images of dorsal skin lesions were captured to dynamically record disease progression at predetermined time points, days 1, 7, 15, and 22 for the DNCB-induced model, and days 0, 3, 6, and 9 for the carvacrol-induced model. Lesion severity was assessed according to four clinical indicators: dryness/scarring, erythema/hemorrhage, edema, and excoriation/erosion. Each symptom was graded on a 0–3 scale: 0 = absent, 1 = mild, 2 = moderate, 3 = severe. The total dermatitis score ranged from 0 to 12 [40]. Ear thickness in both AD models was measured using an electronic digital caliper (Meinaite, MNT-048, Shanghai, China). Measurements were performed on days −1, 1, 7, 9, 15, and 22 in the DNCB model, and on days 0, 3, 6, and 9 in the carvacrol model. Spleen tissues were dissected, rinsed with 0.9% sterile saline, weighed with an electronic balance, and photographed using a camera (Sony Group Corporation, Tokyo, Japan) for morphological observation.

### 4.4. Histopathological Analysis of Skin Tissue

Mice were euthanized by CO_2_ asphyxiation. Dorsal skin and ear tissues were immediately excised and fixed in 10% (*v*/*v*) formalin. After 24 h of fixation, tissues were dehydrated, paraffin-embedded, and sectioned into 3 μm-thick slices. Hematoxylin–eosin (HE) staining was performed to evaluate epidermal hyperplasia and dermal inflammatory cell infiltration, while toluidine blue staining identified mast cell degranulation. Histopathological scoring was independently performed by two pathologists based on three criteria: dermal inflammatory cell infiltration (eosinophils and mast cells), dyskeratosis (poor keratinization/hyperkeratosis), and epithelial hyperplasia. Each criterion was scored on a 0–3 scale (0 = normal, 1 = mild, 2 = moderate, 3 = severe), with the total score as the sum for the comprehensive assessment [41]. Tissue images were captured under a light microscope, mast cells were counted in 5 random fields per mouse, and histological changes were quantified using ImageJ version 2.14.0 software (NIH, Bethesda, MD, USA).

### 4.5. Immunohistochemistry

After dewaxing and rehydration, antigen retrieval of paraffin-embedded skin tissue sections was performed by microwave heating in EDTA buffer. Endogenous peroxidase activity was blocked with 3% H_2_O_2_. The sections were then blocked with 5% bovine serum albumin before incubation with primary antibodies against β-catenin, COX-2, and TRPV3, which were diluted in PBS at 1:500, 1:500, and 1:800, respectively. Following the 3,3′-diaminobenzidine (DAB) chromogenic reaction, tissue sections were counterstained with hematoxylin, dehydrated, and mounted with permanent aqueous mounting medium for coverslipping. The spectral optical density of the stained sections was measured using a CRi Nuance Multispectral Imaging System (Cambridge Research and Instruments, Woburn, MA, USA), which collected spectral data at 10 nm intervals over a wavelength range of 420–720 nm [42].

### 4.6. Isolation and Culture of Mouse Keratinocytes

Epidermal keratinocytes were isolated from 5- to 8-week-old KM mice. Briefly, the shaved dorsal skin was disinfected with 75% ethanol for 120 s. Under sterile conditions, the subcutaneous tissue and blood vessels were carefully removed. The tissue samples were washed three times with PBS containing penicillin and streptomycin. The samples were then incubated with 1.2 U/mL Dispase II at 4 °C for 18 h. After mechanical separation of the epidermis from the dermis, the epidermis was transferred to 0.25% trypsin-EDTA solution and incubated at 37 °C with gentle shaking for 45 min. Digestion was halted by adding DMEM supplemented with 10% FBS, and the cell suspension was centrifuged at 1000 rpm for 5 min. The resulting cell pellet was collected and resuspended in RPMI-1640 medium supplemented with 1% FBS. The cells were seeded onto culture dishes pre-coated with coverslips and incubated at 37 °C in 5% CO_2_ for 24 h.

### 4.7. Calcium Fluorescence Imaging

Primary cultured keratinocytes were washed three times with PBS, followed by incubation with Fluo-4 AM at 37 °C for 45 min. Unincorporated fluorescent probe was removed by washing cells 2–3 times with PBS, followed by the addition of 900 µL extracellular solution (140 mM NaCl, 5 mM MgCl_2_, 10 mM glucose, 5 mM KCl, and 10 mM HEPES, pH 7.4). Basal intracellular calcium fluorescence intensity was measured first, then 10 µL of each drug treatment was gently added to the culture dish with a micropipette. Calcium fluorescence intensity changes were monitored for 60 s using a laser scanning confocal microscope calcium imaging system (Nikon Nis, Tokyo, Japan). Evaluated treatments included 500 µM carvacrol alone or combined with 30 µM dyclonine, 1.5 µg/mL FOE, 0.72 ng/mL α-pinene, 0.593 ng/mL linalool, or 3.59 ng/mL 1-octanol [43]. Fluo-4 AM was excited at 494 nm with an emission wavelength of 516 nm. Fluorescence intensity changes (F) were normalized to the baseline (F0) and expressed as (F/F0 × 100%) [44].

### 4.8. Statistical Analysis

The data were analyzed by two-way analysis of variance (ANOVA) supplemented with Bonferroni’s multiple comparison test for post hoc analysis, establishing a threshold of *p* < 0.05 for statistical relevance. The quantitative results were graphically represented and statistically analyzed via GraphPad Prism version 8.0.1, with the experimental outcomes expressed as arithmetic means accompanied by their standard errors of the mean (SEMs).

## 5. Conclusions

FOE and its bioactive constituents exhibit reliable protective effects in experimental AD models. The underlying mechanisms are closely attributed to the coordinated regulation of the TRPV3 signaling cascade and β-catenin and COX-2-associated inflammatory responses, which contribute to the amelioration of epidermal pathological damage and the improvement of epidermal tissue structure. These findings provide novel experimental evidence for the pharmacological application of frankincense-derived extracts, supporting FOE as a promising natural candidate for the adjuvant treatment of atopic dermatitis.

## Data Availability

The original contributions presented in this study are included in the article/Supplementary Materials. Further inquiries can be directed to the corresponding authors.

## Supplementary Materials

The following supporting information can be downloaded at: https://www.mdpi.com/article/10.3390/ijms27157061/s1.

## Abbreviations

The following abbreviations are used in this manuscript:

FOE	Frankincense oil extract
AD	Atopic dermatitis
TRPV3	Transient receptor potential vanilloid 3
DNCB	2,4-dinitrochlorobenzene
COX-2	Cyclooxygenase-2
HE	Hematoxylin and eosin
DDE	Diclofenac diethylamine emulgel
