# Supplementary material for: Amelioration of Atopic Dermatitis by Frankincense Oil Extract Is Associated with TRPV3 Regulation and Cutaneous Inflammation Suppression
Source: Int J Mol Sci. 2026 Aug 6;27(15):7061. doi: 10.3390/ijms27157061 (PMC13466975; doi:10.3390/ijms27157061)
Supplement: Supplementary file 1 [file ijms-27-07061-s001.zip › ijms-4453564-supplementary.pdf]

Supplementary Materials

1. Hematoxylin and eosin (H&E) staining

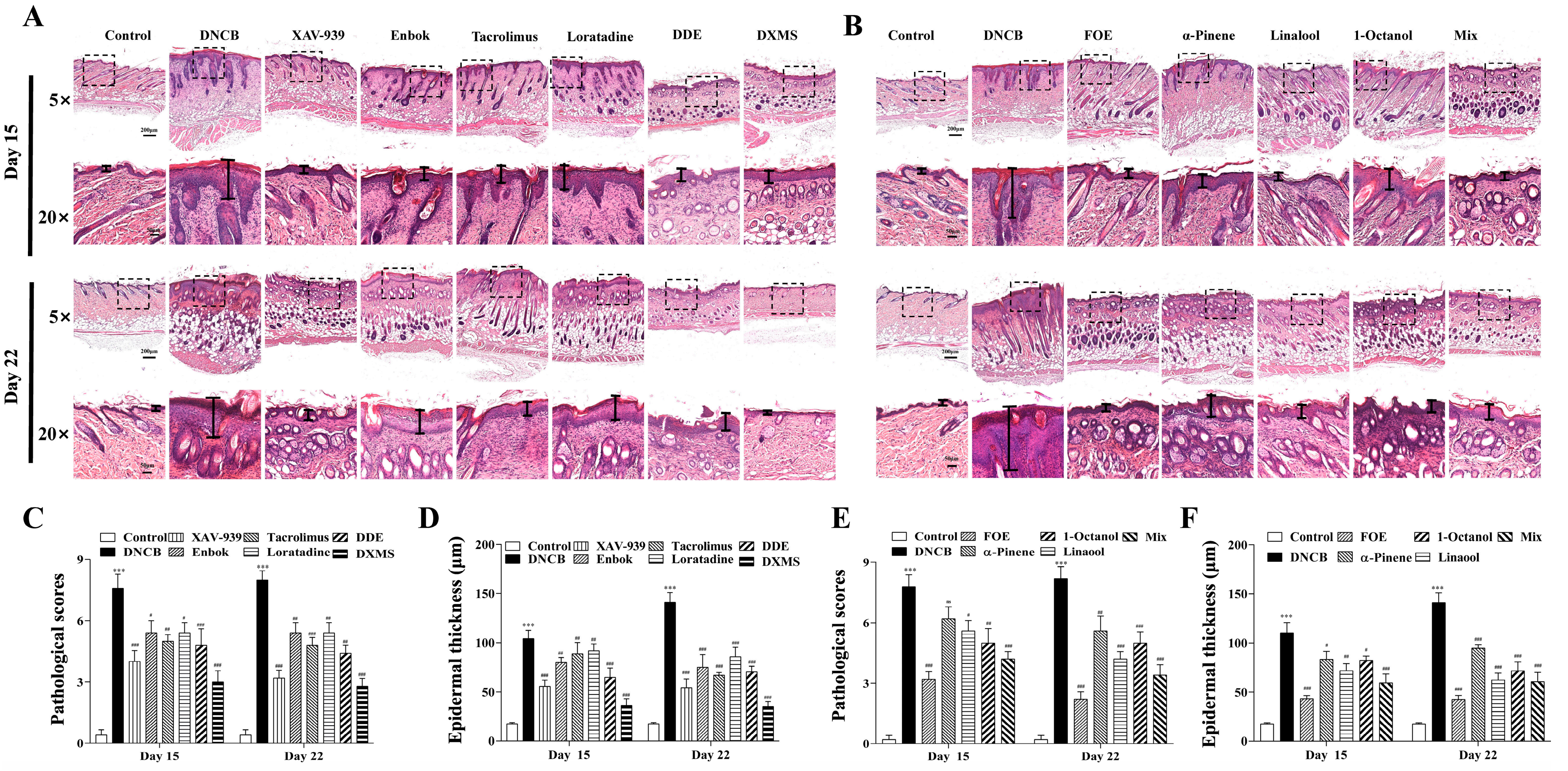

Supplementary Figure S1. The effects of FOE and its active ingredients with clinically used drugs and related inhibitors treatment on the histopathological changes of skin in AD mice. Representative images of HE staining of clinically used drugs and related inhibiting groups (A) and in the FOE and its active ingredient groups (B). Pathological scores (C) and epidermal thickness (D) of clinically used drugs and related inhibiting groups. Pathological scores (E) and epidermal thickness (F) of FOE and its active ingredient groups. The black line segment indicates the epidermal thickness of the dorsal skin. Two-way ANOVA was used to express experimental data as mean ± SEM. Compared to the control, $*p < 0.05$; $**p < 0.01$; $***p < 0.001$. Compared to the DNCB group, $^{\#}p < 0.05$; $^{\#\#}p < 0.01$; $^{\#\#\#}p < 0.001$; ns, not significant. Scale bars represent 200 µm, 50 µm.

Toluidine blue (TB) staining

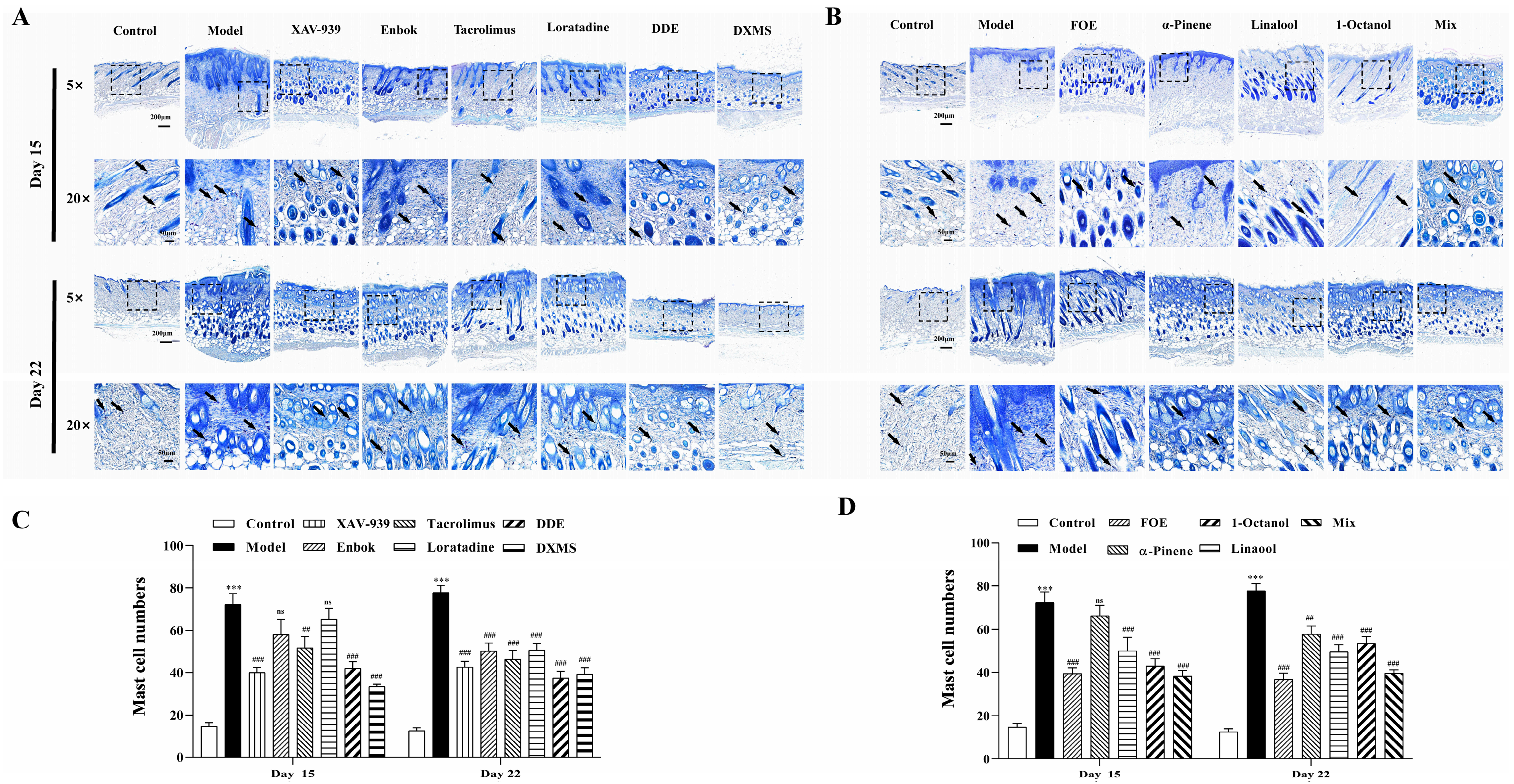

Supplementary Figure S2. The effect of FOE and its active ingredients with clinically used drugs and related inhibitors treatment on mast cells infiltration in AD mice. Representative images of toluidine blue staining of clinically used drugs and related inhibitors groups (A) and in the FOE and its

active ingredient groups (B). Counting of mast cells in the clinically clinically used of drugs and related inhibitors groups (C) and in the FOE and its active ingredient groups (D). The black arrows point to the mast cells. Two-way ANOVA was used to express experimental data as mean ± SEM. Compared to the control, *$p < 0.05$; **$p < 0.01$; ***$p < 0.001$. Compared to the DNCB group, $^{\#}p < 0.05$; $^{\#\#}p < 0.01$; $^{\#\#\#}p < 0.001$; ns, not significant. Scale bars represent 200 μm, 50 μm.
